# Lanthanum-based metal organic framework (La-MOF) use of 3,4-dihydroxycinnamic acid as drug delivery system linkers in human breast cancer therapy

**DOI:** 10.1186/s13065-022-00886-y

**Published:** 2022-11-12

**Authors:** Moosareza Safinejad, Amir Rigi, Malihe Zeraati, Zohreh Heidary, Shohreh Jahani, Narendra Pal Singh Chauhan, Ghasem Sargazi

**Affiliations:** 1 RF MEMS & Micro Bio-Nano Electronics (MBNE) Lab, Department of Electrical Engineering, Shahid Bahonar University of Kerman, Kerman, Iran; 2 Department of Nursing, Young Researchers and Elite Club, Zahedan Branch, Islamic Azad University, Zahedan, Iran; 3 Department of Materials Engineering, Shahid Bahonar University of Kerman, Kerman, 761694111 Iran; 4 Vali-E-Asr Reproductive Health Research Center, Family Health Research Institute, Tehran University of Medical Sciences, Tehran, Iran; 5 Noncommunicable Diseases Research Center, Bam University of Medical Sciences, Bam, Iran; 6 Department of Chemistry, Bhupal Nobles’ University, Udaipur-313002, Rajasthan, India

**Keywords:** Synthesis, Metal organic framework, Linker, Characterization, Drug delivery, Anticancer

## Abstract

Metal organic frameworks (MOFs) have received a lot of attention in the research community due to their unique physical properties, which make them ideal materials for targeted drug delivery systems. In this paper, we describe the synthesis of a non-toxic La-based MOF with 3,4-dihydroxycinnamic acid (3,4-DHCA) as a linker. Scanning electron microscopy (SEM), transmission electron microscopy (TEM), energy dispersive spectroscopy (EDS), fourier transform infrared (FTIR) spectroscopy, thermogravimetric analysis (TGA), nitrogen adsorption–desorption measurements, and X-ray powder diffraction (XRD) have all been used to characterize it thoroughly. The La-based MOF showed good biocompatibility with the human breast cancer cell line MDA-MB-468. The ability of 3,4-DHCA to treat MDA-MB-468 cells was confirmed by 40.35% cell viability with La-based MOF. Based on the findings, La-based MOF can be recommended as a promising candidate for anticancer delivery.

## Introduction

A group of lattice solids characterized by crystallization and porosity are called metal–organic frameworks (MOF), and major research has been done on the shape, surface, and pore measurements [1, 2]. The MOF compounds are the result of the binding of metal cations by organic ligands and are used in various fields, including drug delivery [3], gas storage [4], luminescent [5] and anticancer [6]. Due to the adjustable construction and chemical properties that change and function, MOF has many advantages over other drug delivery systems [7–9]. However, the high porosity and large surface area of MOFs have resulted in high loading and adsorption capacity, so poor coordination interaction ensures the biodegradability of MOFs [10]. MOFs have a major impact on colloid stability, cell uptake, and drug release profiles, [11, 12]which is highly desirable for pharmaceutical applications due to its surface properties and size [13]. MOFs have anti-cancer properties, for example Cu-MOF [14], Al-MOF [15], Sr-MOF [16], Co-MOF [17], Zn-MOF [18] and Zr-MOF [19] mentioned. Several research articles have recently reported on MOF and MOF-derived nanocomposites as gas sensor [20–22]. Nguyen and coworkers developed porous zeolitic imidazolate frameworks for fluorescent chemosensors [23], and the same group developed Zr-MOF as a nano adsorbent to remove toxic organic dye for water remediation [24]. Dang and coworkers developed Hf-MOF and Fe(III)-MOF using a one-pot three-component reaction with high porosity and surface area for catalytic applications [25, 26].

3,4-Dihydroxycinnamic acid contributes to plant defence mechanisms against predators, pests, and infections by inhibiting the growth of insects, fungi, and bacteria and promoting the protection of plant leaves against ultraviolet radiation B (UV-B) [27, 28]. CA and its derivatives have been studied in vitro and in vivo, and numerous physiological effects, such as antibacterial [29, 30], anti-hepatocarcinoma (HCC) [31], antioxidant [30, 32, 33], anti-inflammatory [30, 32, 33], anticancer [32, 33], and anti-hepatocellular carcinoma activity [34] have been demonstrated. HCC (hepatocellular carcinoma) is one of the leading causes of cancer death worldwide, hence anti-HCC activity is essential [35]. As a result, additional research on the chemical and pharmacological properties of CA is required to contribute to the future development of a novel medicine and, as a result, the extension of therapeutic possibilities [31]. Recently, Rao and coworkers have reviewed the development of MOFs for both in vivo and in vitro drug delivery application [36]. Chemodynamic therapy (CDT) and chemo photodynamic therapy (CPT), which inhibits tumors cell growth and division, are recognized as a potential pharmacological technique used to treat cancer [37, 38]. Tan and coworkers evaluated and discussed various MOFs for their CDT use [39]. Table 1 lists various MOFs and their preparation routes, crystallite size, BET surface area, and action on cell lines.

**Table 1:** MOFs and their anticancer activities on various cell lines

MOFs	Methods	Particle size; surface area	Cell line	Refs.
Cu-MOF	Ultrasonic assisted reverse micelle	less than 100 nm; 284.94 m^2^/g	MCF-7	[18]
Y-MOF	Microwave and ultrasonic irradiation	25 nm, 1267 m^2^/g	MCF-7	[40]
Doxorubicin@Zr-MOF)	One-pot conventional synthesis	–	MCF-7 and HepG-2	[41]
Zeolitic imidazolate crystal framework	One-pot conventional synthesis	250 nm; 495.199 m^2^ g − 1	MCF-7	[42]
Nano Zr-MOF	One-pot conventional synthesis	less than 100 nm; 2296 m^2^ /g	MCF-7/ADR cell and CD44	[43]
5-fluorouracil loaded Cu-MOF	One-pot conventional synthesis	281 nm, 628 m^2^ /g	A549 and HeLa	[44]
Co-MOF	Solvo-thermal synthesis	less than 100 nm; 865 m^2^ /g	HepG, CHO, HeLa, BEAS-2B, MDA-MB-231 and NCI-H460	[45]

Lanthanum has recently been reported as an effective drug for the treatment of malignant tumors. For example, in human cervical carcinoma cells, lanthanum complex has shown significant cytotoxicity in the laboratory [45–47]. LaCl_3_ also reduced the growth and triggered apoptosis in leukemia cell lines, HL-60 and NB4 [48]. Furthermore, lanthanum has been linked to the inhibition of human stomach cancer cells' proliferation [49]. All of these findings revealed that lanthanum, a rare earth element, has the ability to regulate tumor growth [50].

In this study, lanthanum-MOFs (La-MOFs) with linkers of 3,4-dihydroxycinnamic acid (3,4-DHCA) was prepared with a ultrasonic reverse micelle assisted. it was characterized using XRD, TEM, EDS and TGA. It was also tested for anticancer properties.

## Experimental

### Materials and apparatus

Sigma-Aldrich Corporation was chosen to purchase C_12_H_25_NaSO_4_ (sodium dodecyl sulfate) and 8 mL of C_6_H_14_. Merck & Co., Inc. was selected to prepare La(NO_3_)_3_∙6H_2_O (lanthanum nitrate hexahydrate) and C_9_H_8_O_4_ (3,4-dihydroxycinnamic acid).

Each material has been of analytical grade with the increased purity. The Iranian Biological Resource Center (IBRC; Tehran, Iran) provided a human breast cancer (MDA-MB-468) cell line. Trypsin/EDTA solution, 3-(4,5-dimetylthiazol-2-Yl)–2,5–diphenyltetrazolium bromide (MTT), Dulbecco's modified Eagle's medium (DMEM), fetal bovine serum (FBS), phosphate-buffered saline (PBS), and dimethyl sulfoxide (DMSO) were acquired from Gibco BRL and Sigma, respectively. They were developed in DMEM (Gibco, UK) supplemented with 10% FBS (Gibco) and 1% penicillin–streptomycin (Gibco) and incubated at 37 °C in a 5% CO_2_ environment. During the synthesis of MOF, X-ray diffraction (XRD) was used to analyze and determine the crystalline structure and phases. To accomplish this goal, a powder X-ray diffractometer (Expert MPD, pananalytical, CuK = 0.154.6 nm) was employed in the 2θ = 0–50 degree range with a step width of 0.01 degree. The surface morphology was investigated using a scanning electron microscope (FESEM, FEI Nanosem 450). Adsorption/desorption measurements (BET, Belsorp mini II) at 77 K in N_2_ environment were used to assess the porosities, surface area, and pore textural features of samples. Under an Argon environment, thermogravimetric analysis (TGA, TA Q600 USA) was performed from room temperature to 400 °C at a heating rate of 10 °C/min. The cell viability was determined using an ELISA reader (Bio-Tek, Elx 808, Germany) at k = 545 nm.

### Preparation of aqueous extract of *satureja hortensis*

To make *S. hortensis* aqueous extract, combine 25 g of dry Satureja cut (harvested from Golboft in Kerman province of Iran, 2018) in clevenger-type apparatus with 250 mL of distilled water for 1.5 h, then cool and filter three times with centrifugal spinning machine at 3000 rpm. The extract was kept in refrigerator for future uses.

We confirmed that all the relevant permission to collect *S. hortensis* were obtained from the governing body. We are thankful to Prof. Gholamhossein Shahidi Bonjar who undertook the formal identification of the *S. hortensis* used in our study. The voucher ID for this plant was 18/D3713.

### Synthesis of La-based MOF

During the preparation of the specimens using the ultrasonic aided approach, La(NO_3_)_3_.6H_2_O (Control group: 19.0 g NaCl, 16.0 g KCl, 3.0 g C_5_H_8_NO_4_Na and 1.1 g C_12_H_22_O_11_). and 3,4-DHCA were mixed with 1:1 mmol and dissolved in 21 mL of double distilled water. The resultant solution was mixed with 0.077 mmol sodium dodecyl sulphate and 8 mL hexane. The resulting mixture was then agitated for 1 h at 85 °C. The obtained solution was placed in the ultrasonic device and exposed to ultrasonic irradiation under optimal conditions, which included an ultrasonic time of 21 min, an ultrasonic temperature of 40 °C, and a power of 175 W. After 30 min, La-MOF crystals develop, which are centrifuged and washed with DMF. Using 75% of the ultrasonic power at 80 °C, a very high yield of 92.31% La-MOF was obtained.

### Cytotoxicity

To begin treatment, 5 × 10^3^ cells/well were seeded in 96-well flat-bottomed plates overnight; the cells were then subjected to different doses of the herbal extract (0–100.0 M) and La-based MOF (0–100.0 M) for 48 h. After removing the medium, 200 μL of MTT solution (5 mg/mL in PBS) was applied to each well and incubated for 4 h at 37 °C. Following the removal of the solution, 100 μL of DMSO was added to the plates, which were shaken for 15 min. Using an ELISA microplate reader, the absorbance of each sample was measured at 570 nm. The results were expressed as a percentage of cell viability compared to untreated control cells.

## Results

**Fig. 1:**
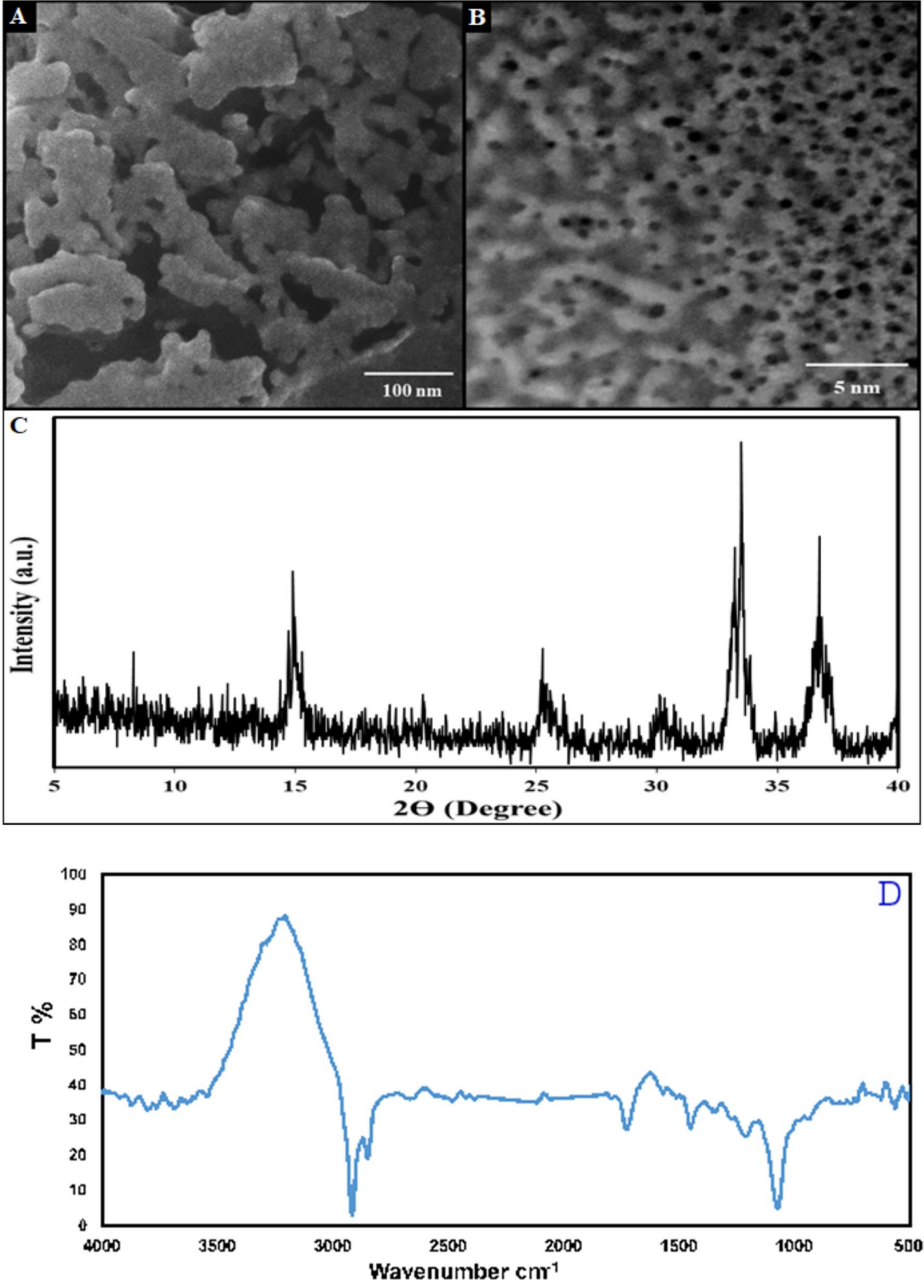
**A** FE-SEM image and **B** TEM image. **C** XRD pattern **D** FT-IR spectrum of the La-based MOF

**Fig. 2:**
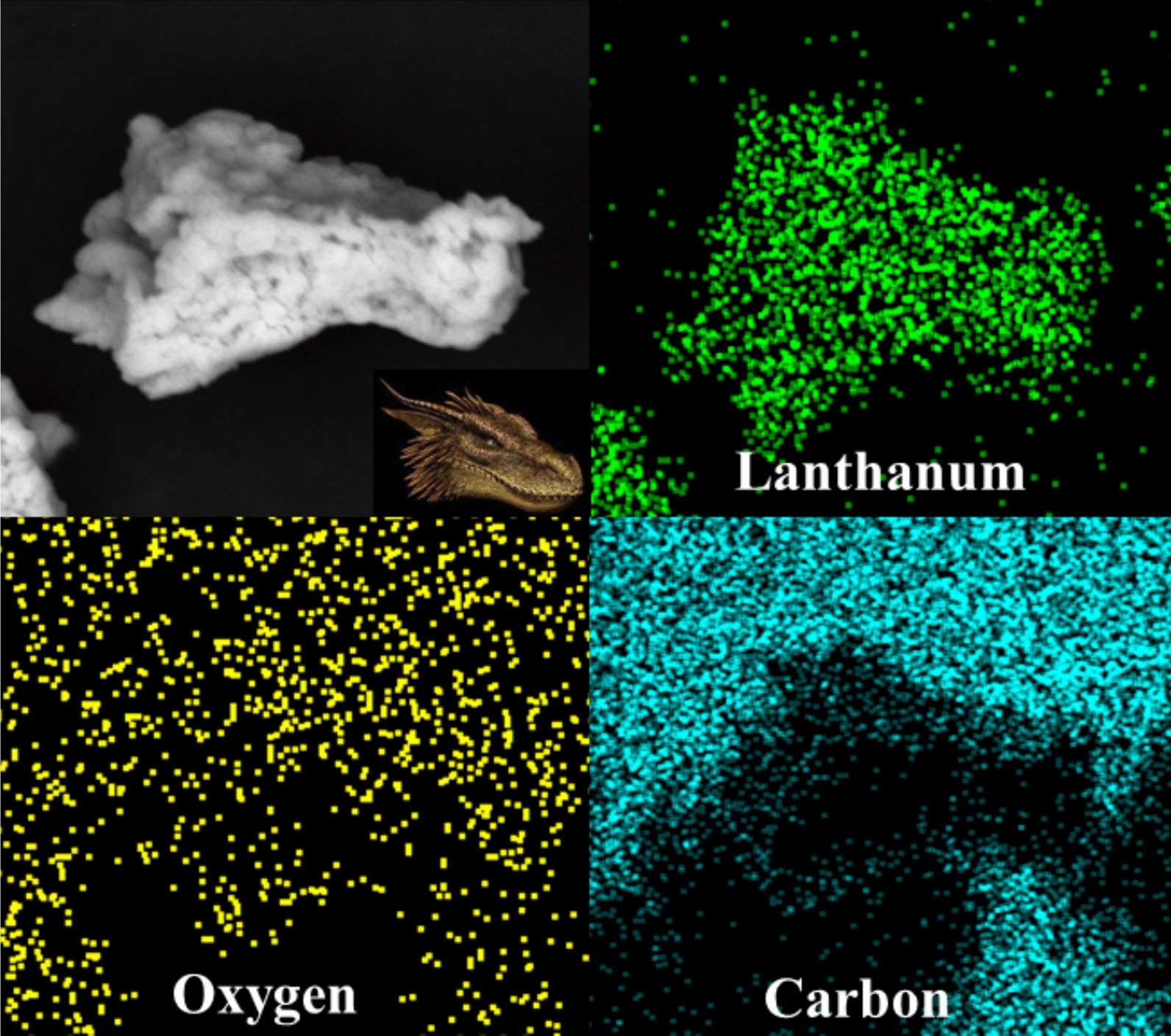
EDX map images of La-based MOF. Inset: Digital photograph of a dragon's head

**Fig. 3:**
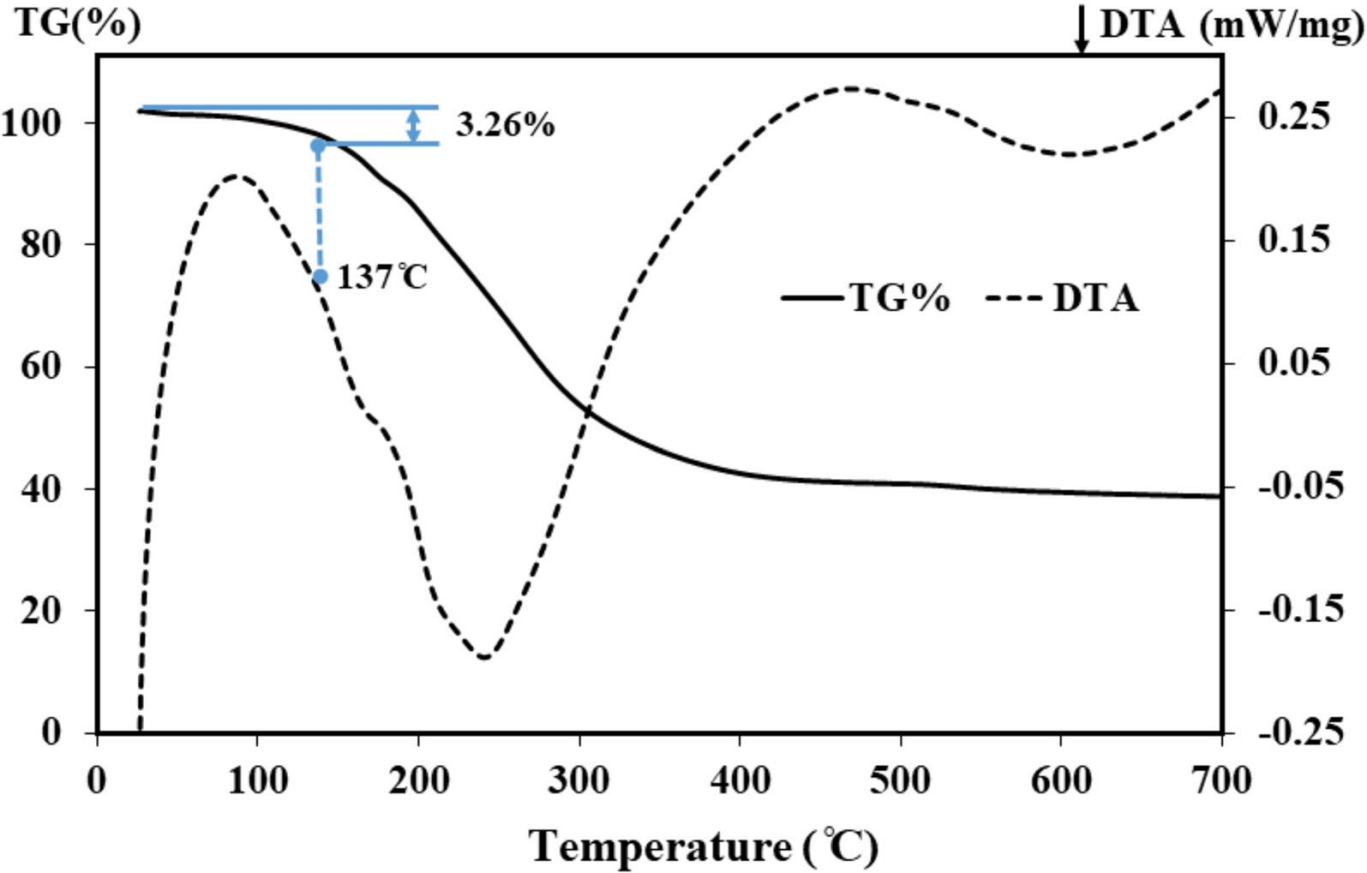
Thermal gravimetric analysis (TGA) and derivative thermogravimetric analysis (DTG) curves of La-based MOF

The surface morphology of the La-based MOF was studied by SEM micrograph (Fig. 1A and B). The crystallite size, phase structure and crystallinity of the La-based MOF were studied by XRD analysis. The XRD spectrum of La-based MOF is shown in Fig. 1C. The thermogravimetric analysis of the La-based MOF was carried out to determine the exact temperature needed for its decomposition. This could be further experimentally confirmed by the EDX elemental analysis of the sample shown in Fig. 2. Figure 3 illustrates the thermal analysis of La-based MOF as a final product. N_2_ adsorption–desorption analysis was used to explore the textural features of La-based MOF.

Figure 5 shows how the MTT test was used to assess the anticancer activity of the La-based MOF and 3,4-DHCA in a human breast cancer cell line in vitro.

## Discussion

### Characterization of the La-based MOF

In the SEM image of La-based MOF can be seen uniform surface morphology (Fig. 1A). Besides, the TEM image of La-based MOF displayed that the surface of La-based MOF was uniform with many cavities in the internal zones. Therefore, this structure is capable to diffuse water into the beads and release the 3,4-DHCA (Fig. 1B).

The seven characteristic diffraction peaks of La-based MOF appeared at 2θ = 14.95°, 20.29°, 25.27°, 30.19°, 33.28°, 35.56° and 36.80° (Reference code: 96–450-4165) (Fig. 1C). No extra-diffraction peak in the XRD spectrum confirms the excellent purity and crystallinity of the La-based MOF. The crystallite size of the synthesized La-based MOF was estimated by Debye–Scherrer formula (Eq. 1).

1 $$\mathrm{D }= \frac{0.9\uplambda }{\beta cos\theta }$$

Here, λ stands for the wavelength of X-ray (1.54056 Å for Cu lamp), θ stands for half of the Bragg diffraction angle and β stands for half of the width of maximum intensity diffraction peak. The mean crystallite size of La-based MOF was 42 nm.

The FTIR spectrum of La-MOF is depicted in Fig. 1D. The La-MOF structure contains surface water, which causes the O–H broad stretching band to appear at 3387 cm^−1^. C–H stretching is responsible for a sharp band at 2911 cm^−1^. The bands shown at 1602 and 1377 cm^−1^ are attributed to the coordinated carboxylate's asymmetrical and symmetrical stretching modes, respectively. According to C–O stretching in La-MOF, a band that developed at 1139 cm^−1^.

As seen in Fig. 2, the EDS mapping images for La-based MOF confirmed uniformly dispersion of each lanthanum, carbon and oxygen elements. If we consider the shape of the MOF as a dragon head, the distributions of the elements are as follows: the scattering of lanthanum is regularly on the dragon head and the carbon is evenly distributed on the background of the dragon head and the oxygen is evenly distributed throughout.

TGA-DTA thermograms for La-based MOF is shown in Fig. 3. La-MOF exhibit a two stage decomposition process. The first stage decomposition starts at 150–204 °C, which may be due to removal of bound water and carboxylate groups as CO_2_ present in the La-MOFs. In the second stage, the main decomposition starts at 270 °C and end up at 450 °C, which corresponds to the loss of side chain attached to the aromatic nucleus. La-MOF exhibits reasonable thermal stability even at high temperatures, as confirmed by TGA and DTA data.

**Fig. 4:**
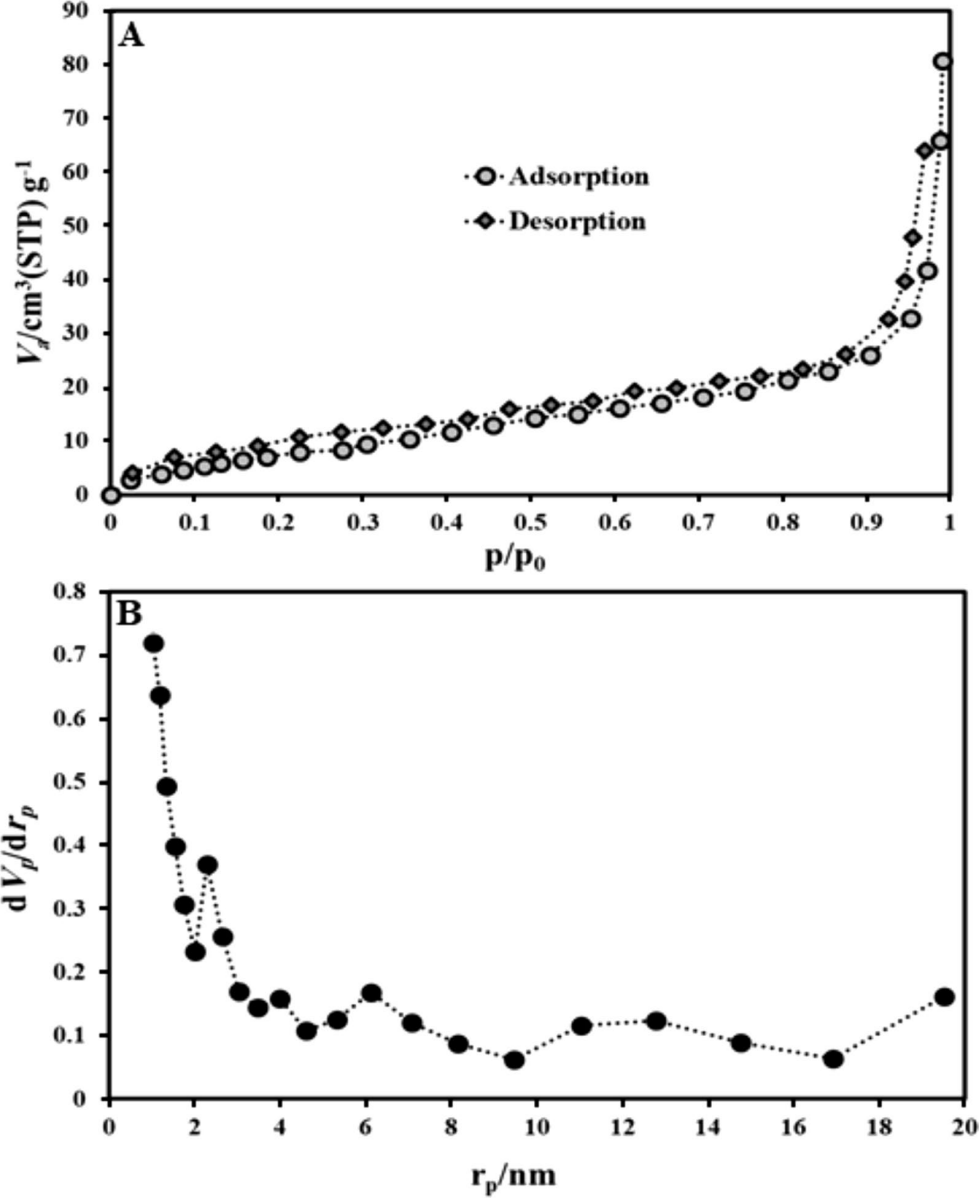
**A** N_2_ adsorption–desorption isotherms La-based MOF; **B** BJH results obtained for La-based MOF

Figure 4a depicts type IV hysteresis loops in the prepared La-based MOF. This isotherm demonstrates the mesoporous structure of the material and the parallel plate shaped pore. The obtained structural parameters such as BET surface area and pore volume of the La-based MOF were 521 m^2^.g^−1^ and 0.075 cm^3^.g^−1^, respectively. Figure 4B illustrates the pore size distribution of the La-based MOF samples on the basis of the Barrett–Joyner–Halenda (BJH) method. This figure also illustrates that the mean pore size distribution of the La-based MOF samples is 8.3 nm, which means the microporous size distributions of the products. It may be an indicative that the La-based MOF remained a wide pore opening and a high porosity, leading to the La-based MOF is one of the best carrier.

### In vitro cytotoxicity studies

**Fig. 5:**
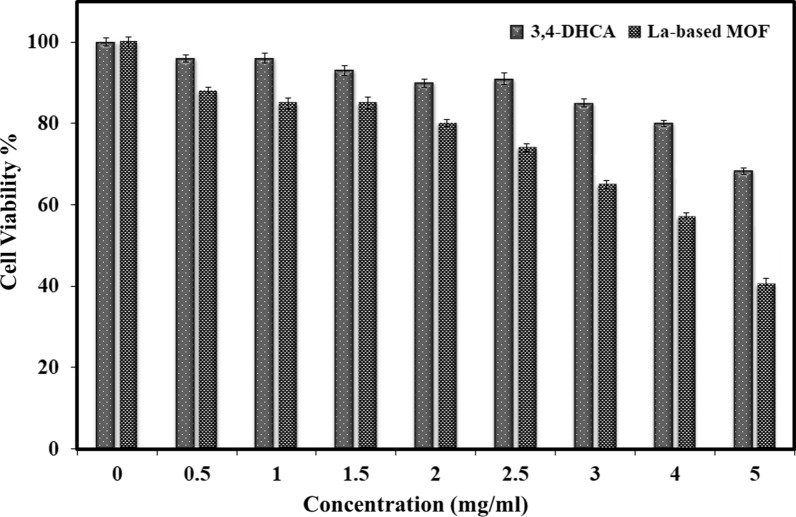
Plots of percentage of cytotoxicity vs. concentrations of the 3,4-DHCA (0–100.0 μM) and La-based MOF (0–100.0 μM) against human breast cancer cells after incubation for 48 h

The values of cell viability found for the La-based MOF and 3,4-DHCA are 40.35% and 68.29%, respectively. Figure 5 shows that the La-based MOF antitumor behavior is greater compared to the 3,4-DHCA. La-based MOF passes through the cell membrane more easily than 3,4-DHCA. Thus, it facilitates the penetration of La-based MOF into cancer cells while improving its antitumor behavior. The anticancer behavior of La-based MOF may be described according to the concept of chelation. The polarity of metal ions may be significantly reduced by chelation because the positive charge is partly shared with the donor groups and also due to the displacement of electrons in the chelate ring. The concentration of metal ions may be exacerbated by the lipophilic properties of chelates and the interaction between the cell wall and metal ions. The geometries and charge dispersion of the La-based MOF molecule are compatible with the cancer cell wall, which increases penetration through the cell wall. Such a unique structural arrangement can break down the cell’s permeability barrier and disrupt the normal process of the cell.

## Conclusion

Summery, in the first step, La-based MOF was prepared and characterized. The preparation of La-based MOF was approved by the SEM, TEM, EDX, TGA, BET and XRD analysis. In addition, in vitro anticancer activity results showed that La-based MOF can inhibit the growth of MDA-MB-468 cells line as a human breast cancer cell. The anticancer activity of the La-based MOF was more than in the 3,4-DHCA. Based upon the results of corresponding experiments, La-based MOF exhibited high 3,4-DHCA drug loading, cancer-targeted release and good biocompatibility, which can be used as a good candidate for anticancer drug delivery system.

## Data Availability

The datasets used and/or analyzed during the current study available from the corresponding author on request. We confirms all the relevant permission to collect sample were obtained from governing body.
